# Late Onset and Protracted Course of Steroid Refractory Chronic Graft-versus-Host Disease

**DOI:** 10.1155/2015/692175

**Published:** 2015-11-03

**Authors:** Gursel Gunes, Haluk Demiroglu, Hakan Goker, Umit Yavuz Malkan, Eylem Eliacik, Okan Yayar, Yahya Buyukasik

**Affiliations:** ^1^Department of Hematology, Hacettepe University Medical School, 06100 Ankara, Turkey; ^2^Department of Hematology, Diskapi Research and Training Hospital, 06100 Ankara, Turkey

## Abstract

Chronic graft-versus-host disease (cGVHD) is one of the most important causes of morbidity and mortality after allogeneic hematopoietic stem cell transplantation (aHSCT). Occurring in 30% to 70% of patients, cGVHD has a median time to onset of 4 to 6 months and most cases present within 2 years after aHSCT. Here, we present a patient transplanted at the age of 55 who developed refractory cutaneous cGVHD more than 5.5 years after aHSCT.

## 1. Introduction

Chronic graft-versus-host disease (cGVHD) is a serious long-term complication which is an important cause of morbidity and mortality after allogeneic hematopoietic stem cell transplantation (aHSCT). Although reduced-intensity-conditioning (RIC) regimen has decreased early transplantation related morbidity and mortality, little success has been achieved for the late term complications [1]. Developing in about 30% to 70% of patients, average time for the onset of cGVHD is about 4–6 months and most cases present within 2 years; onset after 3 years is extremely rare [2]. The risk factors for developing cGVHD are having acute GVHD previously, older age of the patient, usage of peripheral blood as a stem cell source, and treatment with donor lymphocyte infusion. The most commonly affected organ with cGVHD is the skin; erythema and sclerosis are frequent skin manifestations [3]. Here, we aimed to present a skin cGVHD case that developed at the end of 5 years and 7 months after transplant which was refractory to treatment with steroid and other immunosuppressive drugs, photo sensitizer 8-methoxypsoralen ultraviolet A (PUVA), and photopheresis.

## 2. Case Report

A 55-year-old male patient with acute myeloid leukemia (AML-M2) underwent RIC aHSCT in 2004 from his full matched male sibling. He had had methotrexate for four doses (days 1, 3, 6, and 11) and cyclosporine A (CyA) as GVHD prophylaxis. At the end of the third month, he had elevated liver enzymes. A biopsy revealed acute hepatic GVHD. Steroid was added to continuing CyA therapy. Because of rising enzyme levels, CyA could never be stopped, but steroid was tapered because of steroid side effects. CyA was continued at 25–50 mg daily doses until the fourth year of aHSCT when liver enzymes returned to the normal range and stabilized; CyA was then stopped. For about 2 years, the patient was free of any cGVHD signs and symptoms. In this period, the patient has been followed up at the outpatient transplantation clinic every month.

**Figure 1:**
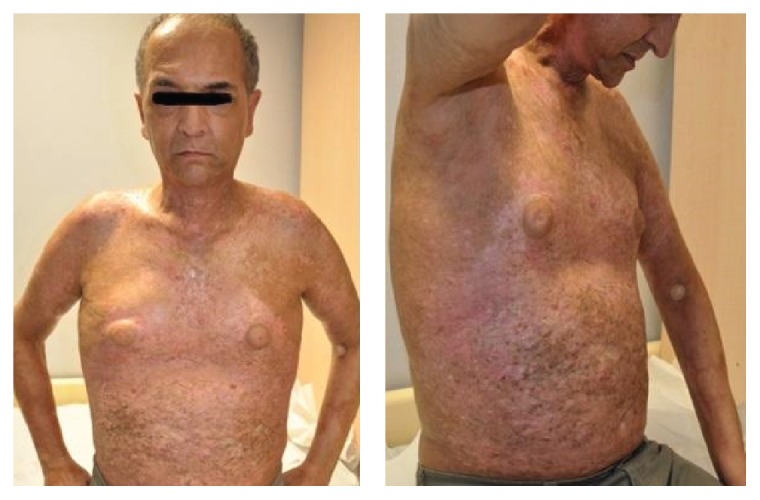
Extensive skin lesions of the patient.

**Figure 2:**
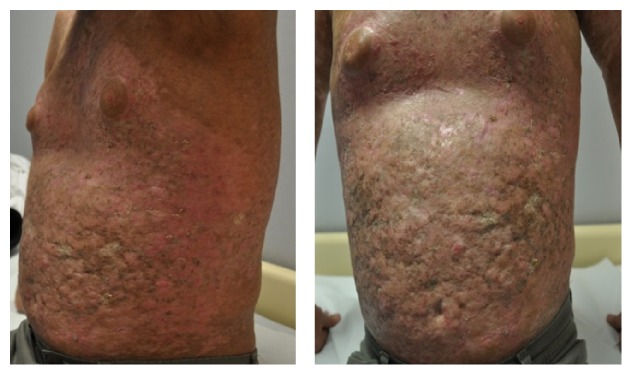
Thickening of the skin.

At about 5 years and 7 months of aHSCT, the patient was admitted to the outpatient transplantation clinic with extensive maculopapular rash and deep sclerotic cutaneous lesions with thickening of the skin in over 50% of the body surface area (Figures 1 and 2). He had no pruritus and no involvement of eye, mouth, and nails. Skin biopsy was reported as lichen sclerosis which was consistent with cutaneous cGVHD. According to the modified organ specific scoring of GVHD in 2014 NIH consensus criteria, the patient had a skin score of 3 for GVHD. This score resulted in making the diagnosis of severe GVHD according to NIH global severity of score [4]. Methylprednisolone 1 mg/kg and CyA 200 mg daily were begun and topical sodium fusidate was applied to skin lesions. Six months later, since the patient had no benefit from that treatment, CyA was stopped, methylprednisolone dose was lowered, and tacrolimus was started at 2 mg per day. As there was no satisfactory response to oral immunosuppressive treatment, PUVA therapy was performed for 22 times in 4 months with oral psoralen. As there was no reducing in the intensity of the lesions, extracorporeal photopheresis was added to the therapy every two weeks for two consecutive days. At about 3 months of phototherapy, skin lesions began regressing. Then, methylprednisolone was stopped and tacrolimus dose was reduced to 1 mg per day. For the last 3 years until May 2015, the patient has been on low dose daily tacrolimus 2 mg and photopheresis once a month. Although regression of the skin lesions was noted during three years, the rate of regression dropped at the end of three years as observed clinically and we decided to administer thalidomide. In June 2015, thalidomide 100 mg per day was started for the still persisting skin lesions. Thus, it has been five years since cutaneous cGVHD was first diagnosed, and limited success has been obtained despite the use of a wide range of therapeutic agents.

## 3. Discussion

cGVHD is one of the major complications of aHCST. The majority of the cGVHD cases present within the first 2 years of aHSCT and 80% of cases have a history of acute GVHD [2]. Average duration of need for immunosuppressant therapy for cGVHD is 2-3 years; however, 10–15% of the patients may have active disease up to 5 years after diagnosis [5].

cGVHD can be seen either limited to one organ system or, more frequently, involvement of two or more organ systems may be present. About half of the patients have lesions of cGVHD of the skin, mouth, and eyes. Lichenoid skin lesions generally occur one year after acute GVHD [6]. Unlike that, our patient developed lichenoid lesions of the skin more than 5.5 years after aHSCT when liver lesions had been in remission for nearly 2 years. The patient was treated with a wide range of immunosuppressive drugs and phototherapy, but limited improvement was observed.

The treatment of cGVHD consists of immunosuppressive drugs such as corticosteroids, CyA, and tacrolimus in the first line. If disease is taken under control with those drugs, the drug doses are decreased and finally they are stopped. However, as in our patient, about half of the patients do not respond to first line therapy. After the first line therapy has failed, we tried with second line treatment modalities. Unfortunately there is no standard treatment strategy in these refractory cases [6, 7]. These patients are at high risk of dying from GVHD or its complications and treatment must be individualized according to the patient's clinical condition [8]. In our case, PUVA, photopheresis, and thalidomide were started sequentially. The patient partially responded. None of them has completely resolved the skin lesions. However, liver cGVHD resolved completely at the end of the fourth year of aHSCT.

It is well known that cGVHD has a strong antileukemic effect [9]. This graft versus leukemia (GVL) effect decreases relapse risk markedly [10]. In cGVHD process, our patient's quality of life was low because of skin lesions but disease did not relapse. This made us think that cGVHD in lower stage that continued for a long time might have protected our patient from relapse of AML with its GVL effect.

In conclusion, cutaneous cGVHD generally develops within the two years after aGVHD and onset after three years is extremely uncommon [2]. However, in our patient, the onset of cutaneous cGVHD is unexpectedly late (5 years and 7 months). More importantly, the patient has never suffered from acute cutaneous GVHD and at the time of the onset of lichenoid and thickening skin lesions there was no sign of cGVHD with complete resolution of hepatic signs 2 years before. This denotes the fact that alloreactivity is not abolished completely after aHSCT.
